# Inhibition of antithrombin by hyaluronic acid may be involved in the pathogenesis of rheumatoid arthritis

**DOI:** 10.1186/ar1487

**Published:** 2005-01-11

**Authors:** Xiaotian Chang, Ryo Yamada, Kazuhiko Yamamoto

**Affiliations:** 1 Laboratory for Rheumatic Diseases, SNP Research Center, The Institute of Physical and Chemical Research (RIKEN), Kanagawa, Japan; 2 Department of Allergy and Rheumatology, Graduate School of Medicine, University of Tokyo, Tokyo, Japan

**Keywords:** antithrombin, glycosaminoglycan, hyaluronic acid, rheumatoid arthritis, thrombin

## Abstract

Thrombin is a key factor in the stimulation of fibrin deposition, angiogenesis, proinflammatory processes, and proliferation of fibroblast-like cells. Abnormalities in these processes are primary features of rheumatoid arthritis (RA) in synovial tissues. Tissue destruction in joints causes the accumulation of large quantities of free hyaluronic acid (HA) in RA synovial fluid. The present study was conducted to investigate the effects of HA and several other glycosaminoglycans on antithrombin, a plasma inhibitor of thrombin. Various glycosaminoglycans, including HA, chondroitin sulfate, keratan sulfate, heparin, and heparan, were incubated with human antithrombin III *in vitro*. The residual activity of antithrombin was determined using a thrombin-specific chromogenic assay. HA concentrations ranging from 250 to 1000 μg/ml significantly blocked the ability of antithrombin to inhibit thrombin in the presence of Ca^2+ ^or Fe^3+^, and chondroitin A, B and C also reduced this ability under the same conditions but to a lesser extent. Our study suggests that the high concentration of free HA in RA synovium may block antithrombin locally, thereby deregulating thrombin activity to drive the pathogenic process of RA under physiological conditions. The study also helps to explain why RA occurs and develops in joint tissue, because the inflamed RA synovium is uniquely rich in free HA along with extracellular matrix degeneration. Our findings are consistent with those of others regarding increased coagulation activity in RA synovium.

## Introduction

Thrombin is a multifunctional protease that can activate hemostasis and coagulation through the cleavage of fibrinogen to form fibrin clots. Increasing fibrin deposition is a predominant feature of rheumatoid arthritis (RA) in synovial tissue, which contributes to chronic inflammation and progressive tissue abnormalities [1]. Thrombin also acts as a mitogen to stimulate the abnormal proliferation of synovial cells during RA pathogenesis. In this regard, thrombin can elevate the expression of nuclear factor-κB, interleukin-6, and granulocyte colony-stimulating factor in fibroblast-like cells of the RA synovium [2,3]. By a similar mechanism, thrombin can upregulate the transcription of vascular endothelial growth factor receptor and thereby induce the permeability, proliferation, and migration of capillary endothelial cells or their progenitors during angiogenesis [4-6]. Thrombin also plays an important role in the proinflammatory process by stimulating neutrophil adhesion to vessel walls and releasing prostacyclin [7]. Thus, thrombin is essential for enhancing synovial thickness and inflammation during the pathogenesis of RA.

The principal plasma inhibitor of thrombin is antithrombin, a single-chain 51 kDa glycoprotein that is synthesized in liver. The inhibitory activity of antithrombin on thrombin is significantly enhanced by heparin, a type of glycosaminoglycan (GAG) [8]. The GAG family comprises large anionic polysaccharides with similar disaccharide repeats of uronic acid and hexosamine. Physiologically important GAGs include hyaluronic acid (HA), chondroitin sulfates, keratan sulfate (KS), heparin, and heparan, which are the major components of joint cartilage, synovial fluid, and other soft connective tissues [9,10]. Along with the destruction of RA joint tissue, a remarkable quantity of various GAG molecules, especially HA, are released from the extracellular matrix of the synovium [9,10], which is a key feature of RA progression. Because GAGs and heparin share a similar molecular structure, we investigated how HA and other GAGs affect antithrombin activity.

## Methods

Highly purified HA, chondroitin sulfate A (CSA), chondroitin sulfate B (CSB), chondroitin sulfate C (CSC), KS, heparin, or heparan (Seikagaku, Tokyo, Japan) were incubated for 24 hours with human antithrombin III at 150 μg/ml (Sigma, St. Louis, MO, USA) at 37°C in working buffer (100 mmol/l Tris-HCl, pH 7.5) containing 5 mmol/l CaCl_2 _or FeCl_3_. The concentration of antithrombin was determined according to its physiologic level in synovial fluid [11,12]. The reaction was stopped with EDTA. Residual activity of antithrombin was analyzed using the chromogenic Actichrome AT III (American Diagnostica, Greenwich, CT, USA) kit, which quantifies antithrombin III activity as follows. After exposure to GAGs, antithrombin was incubated with the thrombin reagent provided with the kit and residual thrombin activity was determined by incubation with the thrombin-specific chromogenic substrate in the kit. Absorbance was measured at a wavelength of 405 nm. Hence, the inhibitory ability of antithrombin on thrombin was inversely proportional to the residual thrombin activity. This assay method is usually used in the clinical setting. We prepared a series of control tests in which HA, CSA, CSB, CSC, and KS were digested in 0.1 mol/l phosphate buffer (prepare 100 ml of the buffer with 94 ml of 0.1 M KH_2_PO_4 _and 6 ml of 0.1 M K_2_HPO_4_, pH 6.2) at 37°C for 2 hours with 0.1 units/ml hyaluronidase (Seikagaku, Japan) before incubation with antithrombin. Hyaluronidase preferentially digests HA rather than other GAGs.

To determine whether HA can prevent heparin from stimulating antithrombin, we simultaneously incubated heparin (10 μg/ml) and various concentrations of HA with antithrombin (150 μg/ml) at 37°C for 24 hours in the presence of 5 mmol/l CaCl_2_. To investigate the effect of HA on antithrombin in the presence of other metal ions, we incubated HA (1 mg/ml) and human antithrombin III (150 μg/ml) at 37°C for 24 hours in the presence of CaCl_2_, FeCl_3_, KCl, MgCl_2_, and NaCl at various concentrations. Residual antithrombin activity was measured as described above.

## Results

**Figure 1:**
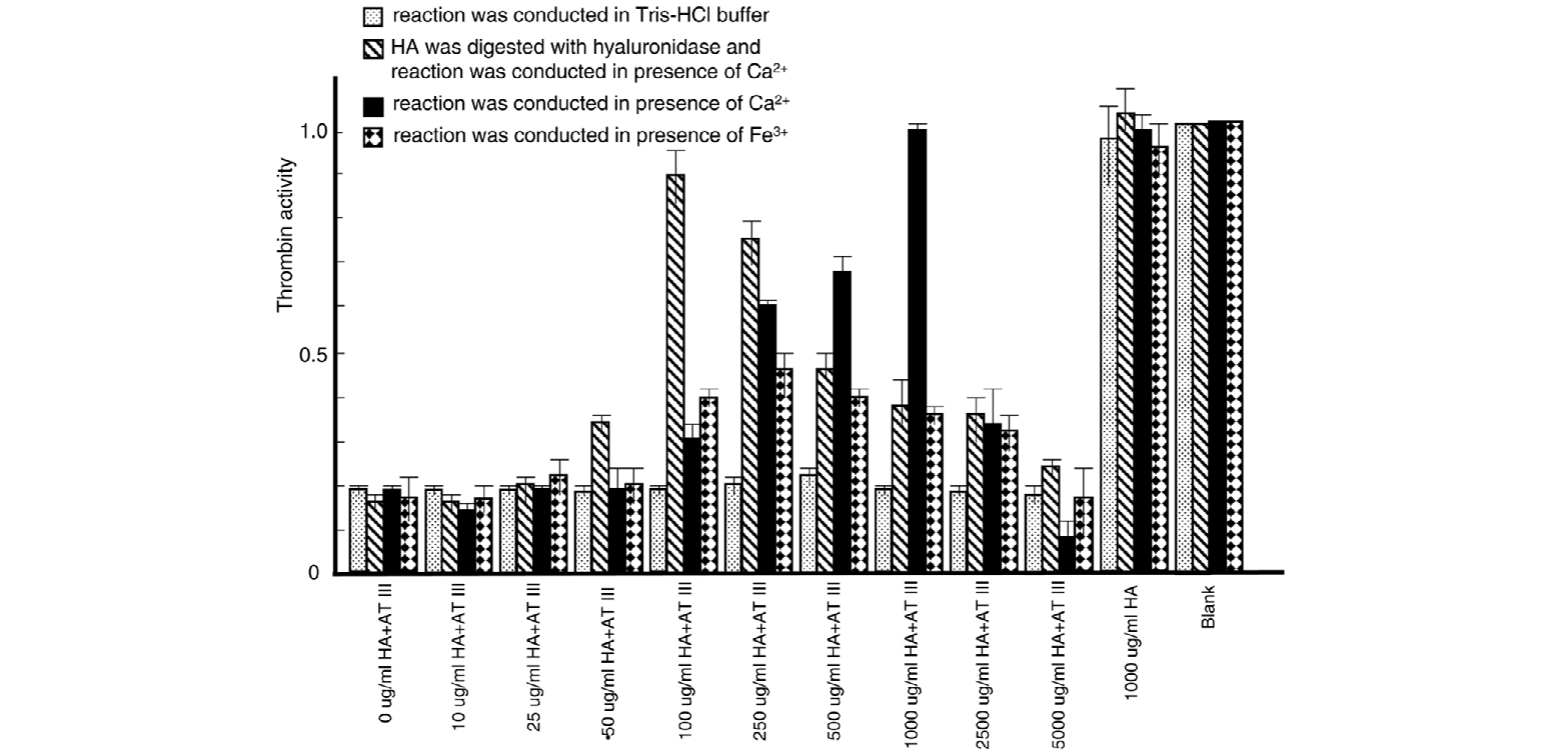
Effect of hyaluronic acid (HA) on antithrombin (AT). Various concentrations of HA, digested or not with hyaluronidase, were incubated with antithrombin in the presence of 5 mmol/l CaCl_2 _or FeCl_3_. Thrombin activity in the absence of both HA and antithrombin (blank) was considered as 1 and the activities of the other tests were normalized based on comparisons with blank. Values are expressed as mean ± standard deviation of data from triplicate experiments.

In the absence of heparin, antithrombin partly inhibited thrombin activity. Low concentrations of HA did not significantly affect antithrombin activity, regardless of the presence or absence of Ca^2+ ^or Fe^3+^. However, HA concentrations above 250 μg/ml considerably suppressed the inhibitory ability of antithrombin against thrombin in the presence of Ca^2+ ^or Fe^3+^, and 1 mg/ml HA completely blocked antithrombin activity under the same conditions. Consequently, thrombin activity was gradually elevated by increasing HA concentrations between 250 and 1000 μg/ml. However, HA at concentrations above 1000 μg/ml progressively lost the ability to prevent inhibition of thrombin activity by antithrombin. Furthermore, HA after digestion with hyaluronidase inhibited antithrombin activity at relatively low concentrations (100 μg/ml) in the presence of Ca^2+^. This observation indicated that the inhibitory effect of HA on antithrombin was not caused by impurities in the reagent. The control without antithrombin indicated that HA does not directly affect thrombin (Fig. 1).

**Figure 2:**
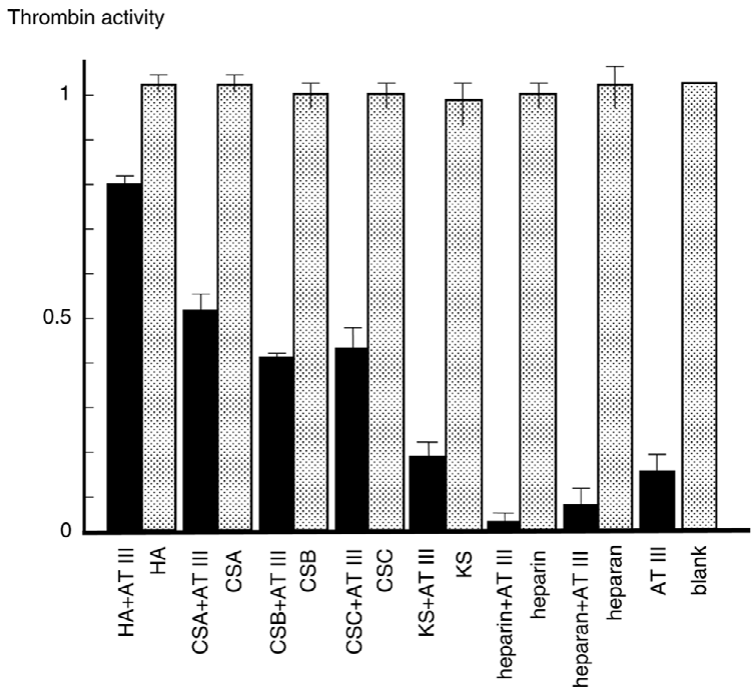
Effects of various glycosaminoglycans (GAGs) on antithrombin (AT). Hyaluronic acid (HA), chondroitin sulfate A (CSA), chondroitin sulfate B (CSB), chondroitin sulfate C (CSC), keratan sulfate (KS), heparin, or heparan (500 μg/ml) was incubated with 150 μg/ml antithrombin and 5 mmol/l CaCl_2_. Controls consisted of only GAG or AT and blank (working buffer only). Thrombin activity of blank was considered as 1 and the activities of other tests were normalized based on comparisons with blank. Values are expressed as mean ± standard deviation of data from triplicate experiments.

**Figure 3:**
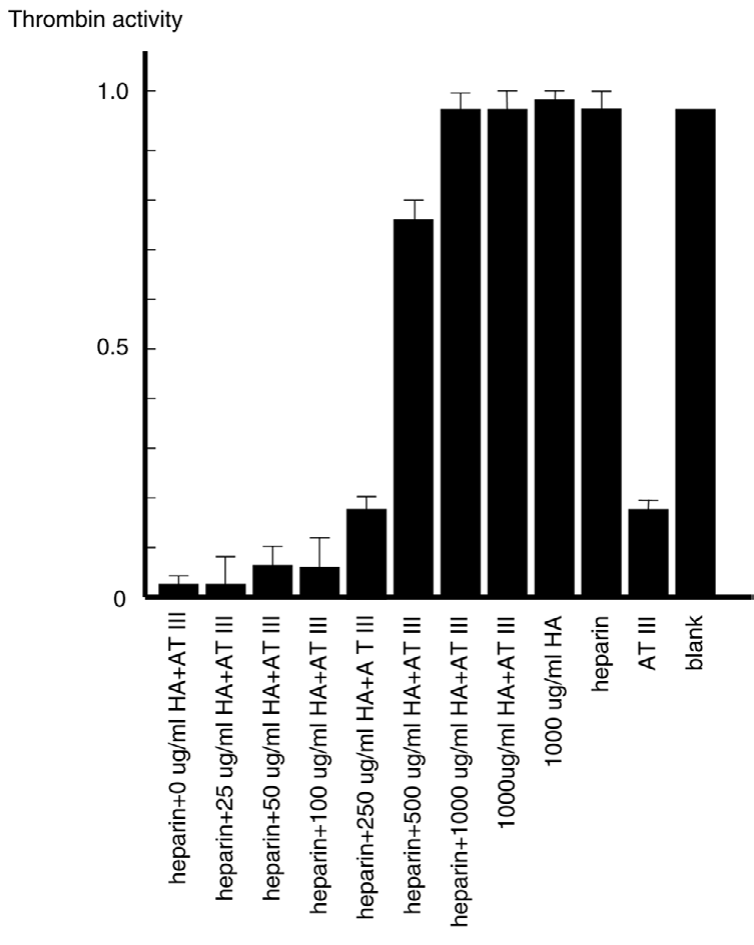
Heparin stimulates antithrombin (AT) activity in the presence of hyaluronic acid (HA). Heparin (10 μg/ml) and various concentrations of HA were incubated with 150 μg/ml antithrombin in presence of 5 mmol/l CaCl_2_. Thrombin activity of blank (reaction buffer only) was considered as 1 and the activities of other tests were normalized based on comparisons with blank. Values are expressed as mean ± standard deviation of data from triplicate experiments.

**Figure 4:**
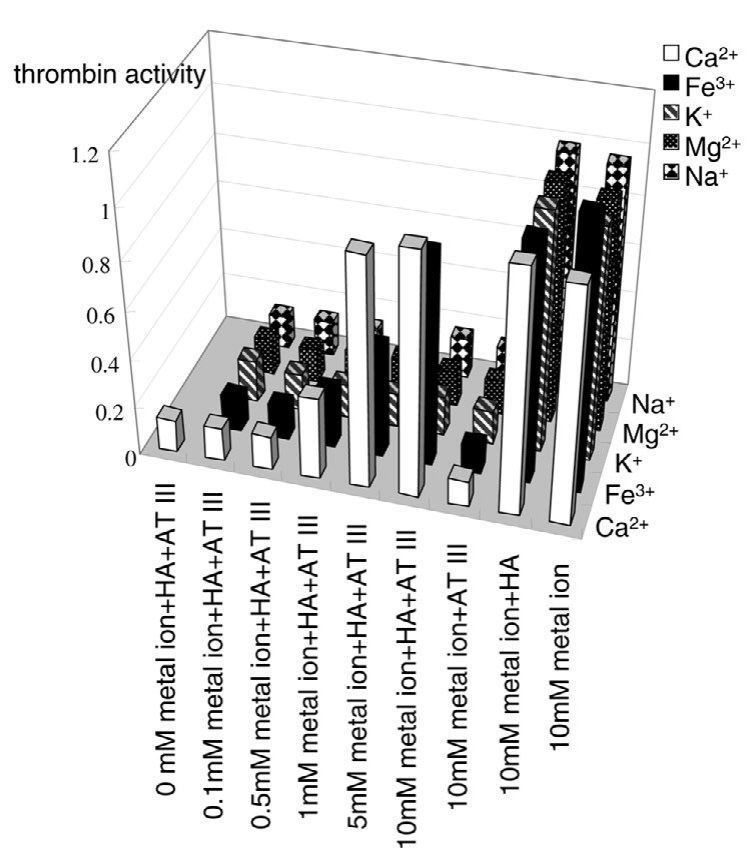
Effects of various metal ions on ability of hyaluronic acid (HA) to inhibit the activity of antithrombin (AT). HA (1000 μg/ml) and antithrombin (150 μg/ml) were incubated with various concentrations of CaCl_2_, FeCl_3_, KCl, MgCl_2_, or NaCl. Thrombin activity of blank (reaction buffer only) was considered as 1 and the activities of other tests were normalized based on comparisons with blank. Values are expressed as mean ± standard deviation of data from triplicate experiments.

CSA, CSB, and CSC also inhibited the antithrombin effect in the presence of Ca^2+ ^but to a lesser extent than did HA (Fig. 2). KS did not significantly affect antithrombin activity. Exposing CSs and KS to hyaluronidase did not clearly change this effect, indicating that CSs themselves inhibit antithrombin (data not shown). In contrast to HA, heparin and heparan clearly stimulated thrombin inhibition by antithrombin (Fig. 2). However, the stimulatory effect of heparin was considerably decreased in the presence of HA and Ca^2+^. Moreover, the ability of HA to prevent heparin activity was progressively strengthened with increased concentrations of HA within the range 250–1000 μg/ml (Fig. 3). Other metal ions, including K^+^, Mg^2+^, and Na^+^, did alter the effect of HA on antithrombin (Fig. 4).

## Discussion

The destruction of joint tissue is a primary feature of RA. In the inflamed RA synovium, proliferating macrophages and colonizing lymphocytes, together with persistent angiogenesis, produce large amounts of matrix metalloproteinases that destroy the surrounding cartilage and extracellular matrix of connective tissue [13]. Because GAGs are the basic structural components of joint cartilage, synovial fluid, and soft tissues [9,10], the RA synovium produces an abundance of free GAGs during tissue destruction. Among these, HA is a predominant component of the articular surface and synovial fluid, in which the HA concentration is between 1500 and 2500 μg/ml [14,15]. Pitsillides and coworkers [14] found that the ratio of free HA to bound HA was significantly increased in the RA (4.53 ± 0.40) as compared with the healthy (1.87 ± 0.42) synovium, although the total concentration of hyaluronan was not increased in the rheumatoid synovium. Their histochemical staining also showed that hyaluronan was concentrated in the lining layer of noninflamed synovial membrane but was more uniformly distributed throughout rheumatoid samples. On the other hand, the HA level is very low among various other tissues. For example, the concentration of serum HA from healthy individuals averages 16 ng/ml, which is 1 × 10^5 ^fold lower than that in synovial fluid [16,17].

The present study found that HA at concentrations between 250 and 1000 μg/ml significantly blocked the ability of antithrombin to inhibit thrombin. This finding helps to explain why RA occurs and develops in joint tissue, because the inflamed RA synovium is uniquely rich in free HA and other GAGs, along with extracellular matrix degeneration. Although the HA levels are higher in RA than in healthy sera [18], we demonstrated that the relatively low levels of HA do not prevent antithrombin activity and thus cannot cause blood clots in the circulation. Hence, only the conditions in the RA synovium can drive the pathogenesis of thrombin-related RA, which includes abnormal angiogenesis, extreme proliferation of fibroblast-like cells, excessive fibrin deposition, and proinflammatory processes. Thus, thrombin-related RA worsens because of the snowball effect of HA release in inflamed joints.

Our notion is supported by many other studies. Jones and coworkers [11] found that antithrombin activity is selectively depressed in RA synovial fluid as compared with that in osteoarthritis, although the concentration of the antithrombin–thrombin complex was significantly increased. Ohba and coworkers [12] also found high levels of thrombin activity in RA synovial fluid. These findings support the notion that inhibiting antithrombin activity plays an essential role in RA pathogenesis. Wang and coworkers [10] recently constructed a model of arthritis by injecting various GAGs into mice. We postulate that the injected GAGs significantly disrupted the inhibition of thrombin by antithrombin, which therefore caused connective tissue disease through abnormally activated angiogenesis, proinflammatory processes, and fibrin deposition. On the other hand, heparan, which has an almost identical structure to that of heparin but contains fewer sulfates, stimulated antithrombin activity in a similar manner to heparin. These observations indicate that the diverse effects of GAGs on antithrombin are due to differences in their molecular configurations. Heparin pentasaccharide can form complexes with antithrombin and expose a reactive proteinase binding loop on the protein surface [19,20]. Because the molecular structure of HA is analogous to that of heparin, HA might exert its effect by binding to the heparin-binding region of antithrombin. However, such binding did not stimulate the activity of antithrombin as did heparin and heparan; in fact, it blocked the ability of antithrombin to inhibit thrombin. In the present study, the stimulatory effect of heparin on antithrombin was considerably decreased in the presence of HA, supporting the notion that HA could compete with heparin for the heparin-binding region of antithrombin.

Remarkably, HA affected the inhibition by antithrombin only within the range 250–1000 μg/ml. At concentrations above 2000 μg/ml, HA either lost its inhibitory effect or elevated the ability of antithrombin to inhibit thrombin. The physiologic level of free HA in the RA synovium is just within the range 500–1000 μg/ml [14]. Some clinical studies have shown that injecting HA into articular rheumatoid joints can ameliorate inflammation [21,22]. Although further investigation is required to elucidate the exact mechanism by which HA inhibits antithrombin, the results of the present study do not refute the notion that optimal proteoglycan uptake can improve overall articular function in patients with arthritis.

Why HA inhibited antithrombin more after than before hyaluronidase digestion remains obscure. Perhaps the small HA molecule can easily bind and thus exert a more inhibitory role on antithrombin. Nagaya and coworkers [23] found high hyaluronidase activity in the synovial fluid and serum of RA patients, implying an abundance of small HA molecules in the RA synovium. Maneirio and coworkers [24] reported that HA at various molecular weights had different effects on the interleukin-1 induced synthesis of both nitric oxide and prostaglandin E_2 _in chondrocytes. How Ca^2+ ^and Fe^3+ ^are involved in inhibiting antithrombin by HA is also poorly understood. Some investigators found that Ca^2+ ^dramatically promotes the ability of heparin to drive antithrombin activity [8,25,26]. Thus, both Ca^2+ ^and Fe^3+ ^ions might play similar roles in HA-induced changes in the configuration of antithrombin.

Synovial fluid from RA patients contains a far greater abundance of free iron than that from patients with osteoarthritis [27,28]. It was reported that Fe^3+ ^stored in the RA synovium perpetuates inflammation by supporting the production of oxygen radicals and by promoting hyaluronic acid degradation, as well as the release of lysosomal enzymes [29]. Telfer and coworkers [30] recently found that proinflammatory cytokines produced in the RA synovium increased the accumulation of iron in synovial fluid. On other hand, Davies and coworkers [31] reported that neutrophils from synovial fluid and the circulation of RA patients could increase the release of free Ca^2+ ^at inflammatory sites. Caruthers and coworkers [32] also showed that calcium signaling is altered in T lymphocytes from RA patients.

**Figure 5:**
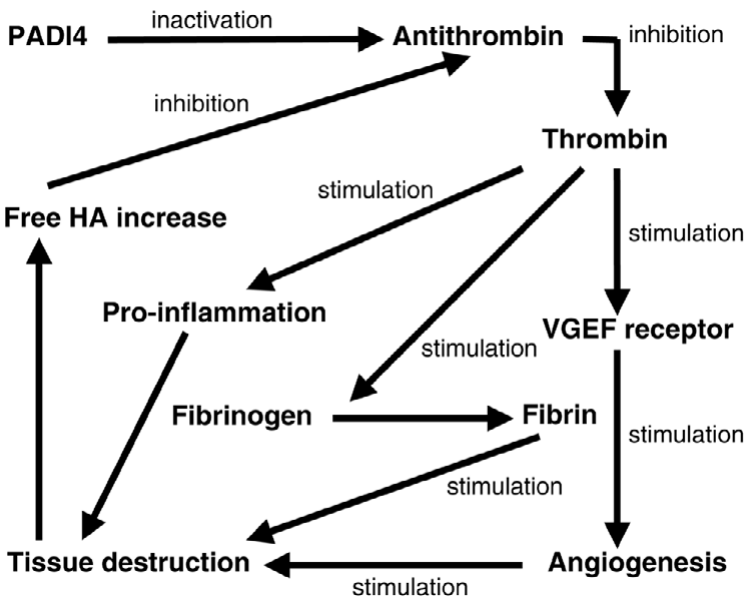
Proposed mechanism of involvement of hyaluronic acid (HA) and peptidylarginine deiminase (PADI4) in the pathogenesis of rheumatoid arthritis. VEGF, vascular endothelial growth factor.

Genome-wide single nucleotide polymorphism analysis has shown that peptidylarginine deiminase (PADI4), an enzyme that post-translationally catalyzes peptidyl arginine to citrulline, is closely associated with RA [33]. We recently found that recombinant human PADI4 protein inactivated human antithrombin III via citrullination *in vitro*. We also detected an increased level of citrullinated antithrombin in the plasma of RA patients [34]. PADI4 is extensively expressed in RA synovial tissue [35,36]. Thus, we suggested that the citrullination of antithrombin is one potential pathway through which PADI4 contributes to the pathogenesis of RA [34]. This notion does not contradict the current findings. We postulate that the genetic, single nucleotide polymorphism-associated disorder of PADI4 and its excessive citrullination of antithrombin play important roles in initiating the RA pathogenic process, whereas inhibition of antithrombin by HA contributes to the development of RA rather than its initiation, because free HA in the synovium achieves high concentrations along with RA progression. Because of abundant Fe^3+ ^and altered Ca^2+ ^metabolism together with significant hyaluronidase activity in the RA synovium, thrombin-related RA specifically worsens in joint tissue as a result of antithrombin inactivation by local PADI4 and free HA (Fig. 5).

HA is an important component of the extracellular matrix. Thrombin and antithrombin play key roles in hemostasis and are involved in the pathogenic processes of many diseases [6,37,38]. The findings presented here should also be useful in investigating the nature of other diseases.

## Conclusion

At concentrations of 250–1000 μg/ml *in vitro*, HA blocked the thrombin-inhibitory ability of antithrombin in the presence of Ca^2+ ^and Fe^3+^. This finding suggested that the high concentration of free HA in diseased RA synovium locally blocks antithrombin under physiologic conditions and thereby deregulates the activity of thrombin. These processes in turn drive the thrombin-related pathogenesis of RA, which includes extensive fibrin deposition, extreme angiogenesis, and abnormal fibroblast-like cell proliferation. Our findings are consistent with those of previous reports regarding increased coagulation activity in the RA synovium.

## Abbreviations

CS = chondroitin sulfate; GAG = glycosaminoglycan; HA = hyaluronic acid; KS = keratan sulfate; PADI = peptidylarginine deiminase; RA = rheumatoid arthritis.

## Competing interests

The author(s) declare that they have no competing interests.

## Authors' contributions

XC designed and executed the study and prepared the manuscript. RY and KY supervised the project, evaluated data, and assisted in preparing the manuscript.
